# Would You Take an Open-Label Placebo Pill or Give One to Your Child? Findings from a Cross-Sectional Survey

**DOI:** 10.2147/PRBM.S439783

**Published:** 2024-02-02

**Authors:** Anne Schienle, Arved Seibel

**Affiliations:** 1 Department of Clinical Psychology, University of Graz, Graz, Austria

**Keywords:** open-label placebos, acceptance, outcome expectation, plausibility, children

## Abstract

**Background:**

Open-label placebos (OLPs), honestly prescribed regarding their inert nature, have been associated with positive health-related effects in both children and adults. However, OLPs are not always perceived by laypeople as a viable treatment option.

**Methods:**

A brief online survey with 806 adult participants (age range: 18–75 years; 29% parents) was conducted to identify predictor variables that are associated with the willingness to take an OLP pill (criterion 1) or to give an OLP to one’s child (criterion 2). The survey covered aspects including the perceived plausibility of the treatment concept for both OLPs and deceptive placebos (DPs), self-reported knowledge about placebos, the expected effectiveness of OLPs in treating emotional/ somatic problems, and attitudes concerning taking pills in general. Multiple hierarchical regressions were carried out.

**Results:**

The expected effectiveness of OLPs in alleviating both emotional and physical ailments and the plausibility of the treatment concepts for both OLPs and DPs significantly predicted the willingness to use OLPs (R^2^ = 0.485). A similar finding was observed when predicting the willingness to administer an OLP to one’s child (R^2^ = 0.443).

**Conclusion:**

Favorable expectations regarding the reduction of emotional and somatic symptoms with OLPs, along with a strong belief in the credibility of placebo mechanisms, play a vital role in influencing the willingness to accept this kind of treatment. These factors can be incorporated into psychoeducational programs.

## Plain Language Summary

Placebos that are administered to a person honestly, without any deception regarding their inert nature, are referred to as open-label placebos (OLPs). Recent research has documented that the concept of OLPs can be subject to skepticism by placebo recipients. Therefore, the current brief survey was directed at identifying factors that are associated with one’s reported willingness to, firstly, take an OLP, and secondly, to treat one’s child with an OLP. The results indicated polarized attitudes towards OLP treatment: a third of the participants held exceedingly unfavorable attitudes regarding OLPs, whereas another third was highly receptive to this form of treatment. Attitudes were influenced by expected treatment effects and the perceived plausibility of placebo concepts.

## Introduction

Placebos that are administered to a person honestly, without any deception regarding their inert nature, are referred to as open-label placebos (OLPs). The use of OLPs circumvents ethical problems associated with conventional (deceptive) placebo treatment, such as the lack of transparency and informed consent. Therefore, the prescription of OLPs would appear to present a promising approach for clinical practice. Indeed, two meta-analyses have revealed positive effects of OLPs in clinical trials on symptom severity in various disorders, such as irritable bowel syndrome, attention-deficit hyperactivity disorder (ADHD), back pain, and cancer-related fatigue.^1^,^2^ Further, a recent meta-analysis of studies that included healthy participants^3^ found significant OLP effects on self-report measures (eg, reduction of emotional distress), but did not find effects for somatic parameters (eg, blood pressure, pain tolerance, wound healing).

Importantly, recent research has highlighted that some placebo recipients approach the concept of OLPs with skepticism, questioning the intuitive logic of taking a pill that is known to not contain any active ingredients.^4–7^ For example, in a study by Haas et al,^4^ participants (taken from the lay population) stated that they found the treatment concept for deceptive placebos (DP) to be more credible than that of OLPs and that they would prefer being treated with a DP instead of an OLP. Further, in a qualitative study by Locher et al,^5^ participants (university students) expressed skepticism concerning the efficacy of OLPs.

Such skeptical attitudes towards OLPs have also been observed in clinical studies. For example, in a study on placebo treatment used as an adjunctive intervention to cognitive-behavioral therapy for patients with depression, 27% of the participants from the OLP group did not return for the follow-up session.^7^ In a subsequent telephone interview, these patients stated that they did not perceive the OLP as being helpful. They were hesitant to disclose this information during the study because they did not want to disappoint the other patients and the therapist.^7^ In the enrollment phase of another trial,^8^ the majority of parents contacted did not consent to their overweight/obese children being administered an OLP treatment for reducing appetite and overeating. Eventually, the study was terminated after a year due to a lack of participants. Other studies, however, have reported positive effects of OLPs (concerning both effectiveness and acceptability) in children with ADHD and functional abdominal pain.^9–11^ In those studies, the OLP was administered as an adjunctive treatment to pharmacotherapy. Moreover, in a survey with pediatric patients and their parents,^12^ 76% of the parents but only 55% of the children reported a positive attitude toward OLP treatment (willingness to take a placebo pill for two weeks to explore if symptom reduction would occur). In sum, the above section illustrates the mixed findings regarding the acceptance and perceived efficacy of OLPs.

Generally, it is seen as favorable that patients understand and choose to accept an intervention in order for symptom reduction to take place. Along these lines, identifying factors associated with a positive attitude towards OLPs may prove important for promoting their use and perceived efficacy.^13^ Healthcare professionals (eg, physicians, physiotherapists, nurses) play a pivotal role in shaping patients’ attitudes and expectations regarding treatment. However, recent surveys have revealed significant variation among these professionals in their understanding and application of contextual placebo factors.^14–17^ This underscores the need for more extensive education for healthcare professionals regarding placebo use as well as standardized placebo practices for ensuring consistent and informed patient care.

The current brief survey focused on potential OLP recipients. It included 806 adult participants (29% parents) and was directed at identifying factors that are associated with one’s reported willingness to, firstly, take an OLP, and secondly, to treat one’s child with an OLP. The survey covered aspects such as the perceived plausibility of the treatment concept for both OLPs and DPs, the self-reported amount of knowledge that participants have about placebos, the expected effects of OLPs on psychological (emotional) and somatic problems, and attitudes concerning taking pills in general. Based on previous OLP studies as well as basic principles of psychoeducation,^4^,^13^ we expected a positive association between these predictors and the two criteria (willingness to take an OLP, willingness to give an OLP to one’s child). This was examined via two multiple hierarchical regression analyses.

## Method

### Sample

A total of 1030 participants returned a questionnaire; 827 data sets were complete. Twenty-one participants had to be excluded (11 participants did not consent to their data being published, three participants reported an age < 18 years. Because the conducted regression analyses included biological sex (male/ female) as a predictor, data from seven participants (reported gender: diverse) were not analyzed due to the small sample size). (A flow chart is depicted in Supplementary Figure S1).

The final sample consisted of 806 participants (mean age *M =* 30.50 years, *SD* = 11.87; 73% female). The level of education in the sample was high: 55% of all participants had a university degree; 5% had less than 12 years of schooling. The majority of participants (85%) indicated no chronic health problems. We additionally analyzed a subsample of 231 parents (mean age of parents: *M* = 42.97 years, *SD* = 13.06; 77% female: mean age of children *M* =10.94 years, *SD* = 11.10), since we were also interested in the attitudes of parents towards allowing their children to take an OLP.

All participants provided written informed consent before taking the survey and agreed to the publication of their anonymized responses. The study complied with the Declaration of Helsinki^18^ and was approved by the ethics committee of the University of Graz, Austria (GZ. 39/26/63 ex 2019/20). Participants did not receive any kind of compensation for participating in the study.

### Procedure

We conducted a web-based cross-sectional observational study with a convenience sample according to the Checklist for Reporting Results of Internet E-Surveys (CHERRIES) guidelines^19^ and to STrengthening the Reporting of OBservational Studies in Epidemiology (STROBE)^20^ (see Supplementary Tables S1 and S2). The online survey was advertised on social media, and through flyers as well as mass mailings at the University of Graz (Austria). The only inclusion criterion was a minimum age of 18 years. Data collection lasted from February 2023 to July 2023.

The online questionnaire consisted of eight statements that had been developed based on questions and comments by parents and children of a previous OLP study.^8^ In the survey, the respondents indicated the degree of agreement for each of the statements included in the survey by moving bar sliders from 0 (no agreement) to 100 (maximal agreement). At the end of the study, participants were able to write comments into a text box (see Supplementary Table S3).

The OLP-related questions of the survey were preceded by a brief introductory text about placebos. This text first defined a deceptive placebo (DP) and provided information about its clinical efficacy.

A placebo pill is a sham treatment that does not contain any active substances (e.g., a sugar pill that looks like a tablet). Patients are not informed that they are receiving a placebo, but instead receive the information that it is a real drug.

Then it was mentioned that DPs cannot be used in clinical practice due to ethical issues and a definition of an OLP was provided (“Patients are told that they will receive a placebo to help relieve their symptoms”). It was also mentioned that it is assumed that OLPs achieve their effects due to previous learning experiences (with medications) and positive expectations.

### Statistical Analysis

Data were analyzed using IBM SPSS Statistics (version 29.0). We computed Pearson correlations and two hierarchical multiple regression analyses. The dependent variables of the regressions were the willingness to take an OLP (analysis of the total sample; n = 806), and the willingness to give an OLP to one’s child (analysis of the parent sample; n = 231). During the first step, we included the sociodemographic variables (sex (male/female), age) in both analyses and additionally the variable parent (yes/no) in the analysis of the total sample. During the second step, six OLP-related items of the survey were included (perceived plausibility of the treatment concept for both OLPs and DPs, amount of self-reported knowledge about placebos, the expected effectiveness of OLPs in treating emotional/ somatic problems, and attitudes concerning taking pills in general). Metric predictors were mean-centered to improve interpretability.

Testing the assumptions for the multiple regression analyses we found no collinearity among the predictors (variance inflation factors varied between VIF = 1.05 and 2.09). Histograms confirmed the normal distribution of the residuals. Additionally, Breusch-Pagan tests yielded non-significant results for both regression analyses (for the willingness to take an OLP: Χ^2^(1) = 1.72, p =0.189 and for the willingness to administer an OLP to one’s child: Χ^2^(1) = 0.48, p=0.488) indicating homoscedasticity.

## Results

**Figure 1:**
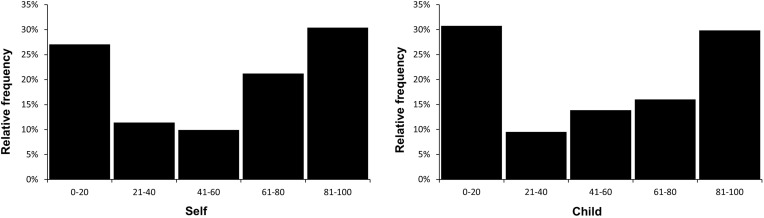
Frequency distributions for the willingness to take an OLP pill (self; n = 806) and to administer it to one’s child (n = 231).

**Note**: scales ranged from 0 to 100 (maximum agreement).

On average, participants were undecided whether they would use an OLP pill for themselves (*M* = 54.9, *SD* = 36.2) and for their children (*M* = 57.5, *SD* = 34.0). Twenty-seven percent of the participants rejected OLP use for themselves (ratings: 0–20), while 31% reported a high willingness to use this type of treatment (ratings: 81–100). A similar divided picture emerged for the willingness to treat one’s children with OLPs (see Figure 1).

The willingness to use an OLP for oneself and one’s children was positively correlated with each other (*r* = 0.64). Moreover, positive associations were found between the belief that OLPs can have positive effects on emotional as well as somatic problems (*r* = 0.64). The perceived plausibility of the OLP and DP concept were only weakly correlated (*r* = 0.10). The plausibility of treating people with DPs was rated significantly higher than the treatment with OLPs (*t*(805) = 23.85, *p* < 0.001; see Table 1).

**Table 1:** Means, Standard Deviations, and Bivariate Correlations Between the Assessed Variables

Variable	M	SD	1	2	3	4	5	6	7	8
1 Willingness OLP self	54.95	36.20								
2 Willingness OLP child	57.37	33.93	0.638**							
3 OLP emotional effect	62.44	30.42	0.558**	0.456**						
4 OLP physical effect	48.54	29.81	0.608**	0.532**	0.635**					
5 Pill avoidance	74.47	25.83	0.173**	0.153**	0.192**	0.187**				
6 OLP plausibility	58.83	32.31	0.603**	0.505*	0.583**	0.604**	0.171**			
7 DP plausibility	80.02	25.22	0.181**	0.264**	0.129**	0.205**	0.154**	0.104**		
8 Placebo knowledge	46.79	34.41	0.088*	0.081*	0.067	0.197**	0.044	0.127**	0.136**	
9 Age (years)	30.50	11.87	−0.141**	−0.145**	−0.068	−0.059	−0.040	−0.140**	−0.032	0.020

**Notes**: n=806; M (means), SD (standard deviations); OLP (open-label placebo); scales ranged from 0 to 100 (maximum agreement); **p < 0.01; *p < 0.05 (two-tailed). 1. Willingness OLP self – “I would take an open-label placebo myself”. 2. Willingness OLP child – “I would support my child taking an open-label placebo”. 3. OLP emotional effect – “I can imagine that open-label placebos can be effective for psychological (emotional) complaints”. 4. OLP physical effect – “I can imagine that open-label placebos can be effective for physical complaints”. 5. Pill avoidance – “I try to solve my own health problems as much as possible without taking tablets/pills”. 6. OLP plausibility – “I find the concept of open-label placebos to be plausible”. 7. DP plausibility – “I find the concept of deceptive placebos to be plausible”. 8. Placebo knowledge – “I already possess prior knowledge about placebos and their effects which goes beyond the information presented here”.

The model of the first regression analysis (criterion: willingness to take an OLP) was significant (*F* (9796) = 83.30, *p* < 0.001). It was found that the plausibility of the OLP concept, the plausibility of the DP concept, and the belief that OLPs can have positive effects on both emotional as well as somatic problems significantly predicted the willingness to take an OLP (see Table 2).

**Table 2:** Hierarchical Multiple Regression Analysis with Willingness to Take an OLP as the Criterion

	R²	B	SE (B)	95% CI (B) [LL, UL]	β	t	p
*Step 1*	0.030						
*Constant*		58.66	1.90	[54.93, 62.39]		30.91	<0.001
*Sex (male, female)*		−7.18	2.87	[−12.81, 1.54]	−0.09	−2.50	0.013
*Age (years)*		−0.25	0.14	[−0.54, 0.03]	−0.08	−1.76	0.078
*Parent (yes, no)*		−6.17	3.77	[−13.57, 1.24]	−0.08	−1.64	0.102
*Step 2*	0.485						
Constant		55.72	1.40	[52.97, 58.46]		39.87	<0.001
Sex (male, female)		−0.57	2.12	[−4.74, 3.60]	−0.01	−0.27	0.789
Age (years)		−0.14	0.11	[−0.35, 0.06]	−0.05	−1.37	0.171
Parent (yes, no)		−2.14	2.78	[−7.58, 3.31]	−0.03	−0.77	0.442
OLP emotional effect		**0.21**	**0.04**	**[0.13, 0.30]**	**0.18**	**5.09**	**<0.001**
OLP physical effect		**0.36**	**0.04**	**[0.27, 0.44]**	**0.29**	**8.06**	**<0.001**
Pill avoidance		0.03	0.04	[−0.04, 0.10]	0.02	0.75	0.453
OLP plausibility		**0.34**	**0.04**	**[0.26, 0.42]**	**0.30**	**8.87**	**<0.001**
DP plausibility		**0.09**	**0.04**	**[0.02, 0.17]**	**0.06**	**2,42**	**0.016**
Placebo knowledge		−0.03	0.03	[−0.08, 0.02]	−0.03	−1.09	0.277

**Note**: n= 806; OLP (open-label placebo); DP (deceptive placebo); unstandardized beta (B); standard error (SE); CI (confidence interval, UL/LL upper/lower limit), standardized beta (β); metric predictors are mean-centered; female sex was coded as 0, male sex as 1; being no parent was coded as 0, having children as 1; bold formatting (statistically significant effects); variable labels represent attitudes as follows: OLP emotional effect – “I can imagine that open-label placebos can be effective for psychological (emotional) complaints”. OLP physical effect – “I can imagine that open-label placebos can be effective for physical complaints”. Pill avoidance – “I try to solve my own health problems as much as possible without taking tablets/pills”. OLP plausibility – “I find the concept of open-label placebos to be plausible”. DP plausibility – “I find the concept of deceptive placebos to be plausible”. Placebo knowledge – “I already possess prior knowledge about placebos and their effects which goes beyond the information presented here”.

The model of the second regression analysis (criterion: willingness to give an OLP to one’s child) was also significant (*F* (8222) = 22.06, *p* < 0.001). It was found that assumed positive effects of OLPs on both emotional as well as somatic problems significantly predicted the willingness to administer an OLP to one’s child. The plausibility of the treatment concepts for both OLP and DP were marginally significant predictors (Table 3).

**Table 3:** Hierarchical Multiple Regression Analysis with Willingness to Administer an OLP to One’s Child as the Criterion

	R²	B	SE (B)	95% CI (B) [LL, UL]	β	t	p
*Step 1*	0.015						
*Constant*		55.80	3.46	[48.98, 62.62]		16.12	<0.001
*Sex* (male, female)		−9.40	5.89	[−21.01, 2.22]	−0.11	−1.59	0.112
*Age (*years)		−0.11	0.19	[−0.49, 0.26]	−0.04	−0.60	0.549
*Step 2*	0.443						
Constant		57.59	2.69	[52.30, 62.89]		21.43	<0.001
Sex (male, female)		−1.70	4.56	[−10.69, 7.30]	−0.02	−0.37	0.710
Age (years)		−0.16	0.15	[−0.45, 0.13]	−0.06	−1.09	0.278
OLP emotional effect		**0.16**	**0.08**	**[0.01, 0.32]**	**0.14**	**2.08**	**0.038**
OLP physical effect		**0.50**	**0.08**	**[0.33, 0.66]**	**0.44**	**6.05**	**<0.001**
Pill avoidance child		0.07	0.08	[−0.08, 0.22]	0.05	0.91	0.366
OLP plausibility		0.15	0.08	[0.00, 0.30]	0.14	1.96	0.052
DP plausibility		0.13	0.08	[−0.02, 0.28]	0.09	1.66	0.098
Placebo knowledge		−0.06	0.06	[−0.18, 0.05]	−0.06	−1.08	0.281

**Notes**: n=231, OLP (open-label placebo); DP (deceptive placebo); unstandardized beta (B); standard error (SE); CI (confidence interval, UL/LL upper/lower limit), standardized beta (β); metric predictors are mean-centered; female sex was coded as 0, male sex as 1; bold formatting (statistically significant effects); variable labels represent attitudes as follows: OLP emotional effect – “I can imagine that open-label placebos can be effective for psychological (emotional) complaints”. OLP physical effect – “I can imagine that open-label placebos can be effective for physical complaints”. Pill avoidance child – “I try to solve health problems of my child as much as possible without tablets/pills”. OLP plausibility – “I find the concept of open-label placebos to be plausible”. DP plausibility – “I find the concept of deceptive placebos to be plausible”. Placebo knowledge – “I already possess prior knowledge about placebos and their effects which goes beyond the information presented here”.

Exploratory, we computed two additional multiple logistic regression analyses to predict the extreme attitudes concerning OLP treatment (criterion: willingness to take an OLP/ give it to one’s child; scores ranging from 0 to 20 were coded as “0”, values from 81 to 100 as “1”). The analyses yielded comparable results as those found in our primary analyses (see Supplementary Tables S4, S5, and Supplementary Material S1).

A total of 33 participants (4%) provided comments in the text box of the survey (see Supplementary Table S3). Half of the statements conveyed skeptical attitudes towards OLPs, with examples such as “In my opinion, placebos only work if you do not know they are placebos” or “In my opinion, open-label placebos lead nowhere”. Other comments delved into personal experiences with deceptive placebos and assumptions about the mechanisms behind placebos.

## Discussion

This study investigated potential factors that influence adults’ willingness to take an OLP pill themselves or to give an OLP to their children. The findings revealed that approximately a third of the participants held exceedingly unfavorable attitudes regarding OLPs, whereas another third was highly receptive to this form of treatment. Thus, extreme attitudes towards OLPs were present on both ends of the spectrum.

A regression analysis revealed that the willingness to use an OLP for oneself could be predicted by the expected beneficial effects of OLPs on both psychological as well as somatic conditions. As such, positive outcome expectations played a pivotal role in the acceptance of OLP treatment. This finding aligns with the results of a large network meta-analysis that included 37 trials with 3021 participants from clinical as well as nonclinical samples.^21^ Positive treatment expectations were also found in that study to be of great importance for OLPs to work. OLP interventions lacking the prior establishment of at least minimal treatment expectations were not effective.

This finding underscores the importance of directing focus toward outcome expectancies, such as an emphasis on the low risks and potential gains of OLP treatment. At the same time, it appears important that expectations are realistic and not overly optimistic. Notably, certain studies have documented instances of “disappointment effects” concerning OLP treatment. In such cases, the anticipated effectiveness of the OLP before the intervention exceeded the perceived effects of the OLP observed after the intervention.^22^,^23^ In other words, instances of expectation violations arose. Generally, patients’ expectations regarding placebo treatment can be either positive or negative and vary in their precision. Further, these expectations can be either confirmed or disconfirmed by the experience of the treatment. In the case that expectations are disconfirmed by the new experience, some individuals adjust their beliefs about placebos accordingly; however, a subgroup remains “cognitively immune” to the new evidence, employing strategies adapted to maintain the previous beliefs and thus remaining steadfast in their original convictions (for an overview of the ViolEx model, which describes the mechanisms of this process, see^24^). Therefore, in a subsequent investigation, it could be advantageous to assess not only expectations concerning placebo treatment but also individuals’ past experiences with it.

The plausibility of the placebo concept turned out to be another important predictor of the willingness to take an OLP pill. An understanding of how both deceptive placebos (DPs) and nondeceptive placebos yield their beneficial effects was found to be relevant for the acceptance of OLP treatment. Our findings here align with a study on the acceptance of OLPs vs DPs conducted by Haas et al.^4^ In that study, the acceptability of placebos was mediated by the credibility of the rationale provided, as well as the expectancy of positive outcomes. In addition, participants in that study found the treatment concept for deceptive placebos to be more credible than that of OLPs. This was also found in the present survey.

Furthermore, research has indicated that not only the acceptance of treatment but also treatment outcomes are linked to the rationale given. For example, one OLP study on pain perception compared two conditions: one with and one without the provision of a rationale.^25^ In the group with a rationale, participants were informed about the effectiveness of placebos for certain symptoms and disorders (eg, pain, Parkinson’s disease, depression), classic conditioning, positive effects of culturally anchored rituals, and self-healing processes. The comparison group received only a brief definition of a placebo. The study demonstrated that pain reduction only occurred when a rationale had been provided.

Based on these findings, recommendations for placebo prescription have been formulated in an expert consensus paper.^26^ Within this paper, it is stated that

Training of clinicians to communicate about placebo and nocebo effects should include an outline of the effects and clinical implications of both placebo and nocebo effects for different conditions as well as the underlying *neurobiological* and psychological mechanisms. (Table 1, point 7; italics inserted by the authors)

In line with this recommendation, explanations of placebo effects based on the underlying brain mechanisms were found to receive the highest plausibility ratings in a study by Smits et al.^27^ Therefore, psychoeducation in the context of OLP treatment should provide information on not only the psychological but also the neurobiological mechanisms of placebo effects.

The second regression analysis carried out for the criterion “willingness to give an OLP to one’s child” identified similar predictors as the first analysis. The best predictor for the approval of OLP treatment for one’s child was the expected potential of the OLP to reduce somatic symptoms. This again highlights the importance of educating OLP recipients about the physiological correlates of placebo-associated changes in well-being. For instance, for explaining OLP effects in the context of pain management (placebo analgesia), information could be provided regarding the activation of brain regions implicated in pain reduction, along with the release of endogenous opioids, after placebo administration.

Regarding the limitations of our study, it is important to acknowledge that we surveyed a sample of European (Austrian) lay people who were predominantly well-educated and female. This restricts the generalizability of our findings to groups with other sociodemographic characteristics (eg, different geographic areas, cultural/ religious beliefs, or educational backgrounds). Another limitation stems from a potential self-selection bias among survey participants, possibly resulting in an overrepresentation of extreme responses regarding the willingness to use OLPs. Additionally, it is worth considering that factors beyond those covered in our brief survey, such as trust in treatment providers^13^ may also influence the decision to embrace OLPs.

Moreover, the respondents of this survey predominantly indicated having no chronic health problems. Attitudes toward OLPs may differ between individuals with or without diagnoses of mental/somatic disorders. Within this context, it has been shown that OLP effects are larger in clinical compared to nonclinical samples.^21^ This difference might reflect variations in expectations and attitudes toward OLPs. Such variations may also be linked to the particular conditions which are treatable with OLPs. In our study, participants were asked about their belief in the efficacy of OLPs for addressing psychological complaints and somatic illnesses. Future surveys could incorporate a more specific categorization of symptoms/ disorders. The inclination to embrace a placebo approach may vary significantly based on specific symptoms, especially when considering pediatric populations.

Finally, it could be explored whether individuals with very skeptical attitudes toward OLP treatment might profit from psychoeducational programs that explain psychological/ neurobiological placebo mechanisms in more detail and foster optimistic yet realistic expectations concerning this form of intervention.

## Conclusion

The current study contributes to the limited body of research on attitudes toward OLPs. Our results indicated polarized opinions concerning taking OLPs or giving them to one’s child. Positive outcome expectations (the expected effectiveness of OLPs in alleviating both emotional and physical ailments) and the perceived plausibility of placebo concepts (for both deceptive/nondeceptive placebos) predicted these OLP attitudes. Given that optimistic expectations surrounding symptom reduction by OLPs, coupled with a strong belief in the credibility of placebo mechanisms, play a vital role in the willingness to embrace such treatments, integrating these factors into psychoeducational programs could prove to be very important for the effective use of OLPs. These programs should target not only patients but also healthcare professionals.
